# Supplementary material for: Causal interplay between lactose intolerance and gut microbiota: a combined bidirectional Mendelian randomization and in vivo validation study
Source: Front Nutr. 2026 Jun 1;13:1803337. doi: 10.3389/fnut.2026.1803337 (PMC13265576; doi:10.3389/fnut.2026.1803337)
Supplement: Supplementary file 1 [file Data_Sheet_1.zip › supplementary materials/Forward/leave one out cross validation/ebi-a-GCST90027853.finngen_R12_E4_LACTONAS.pdf]

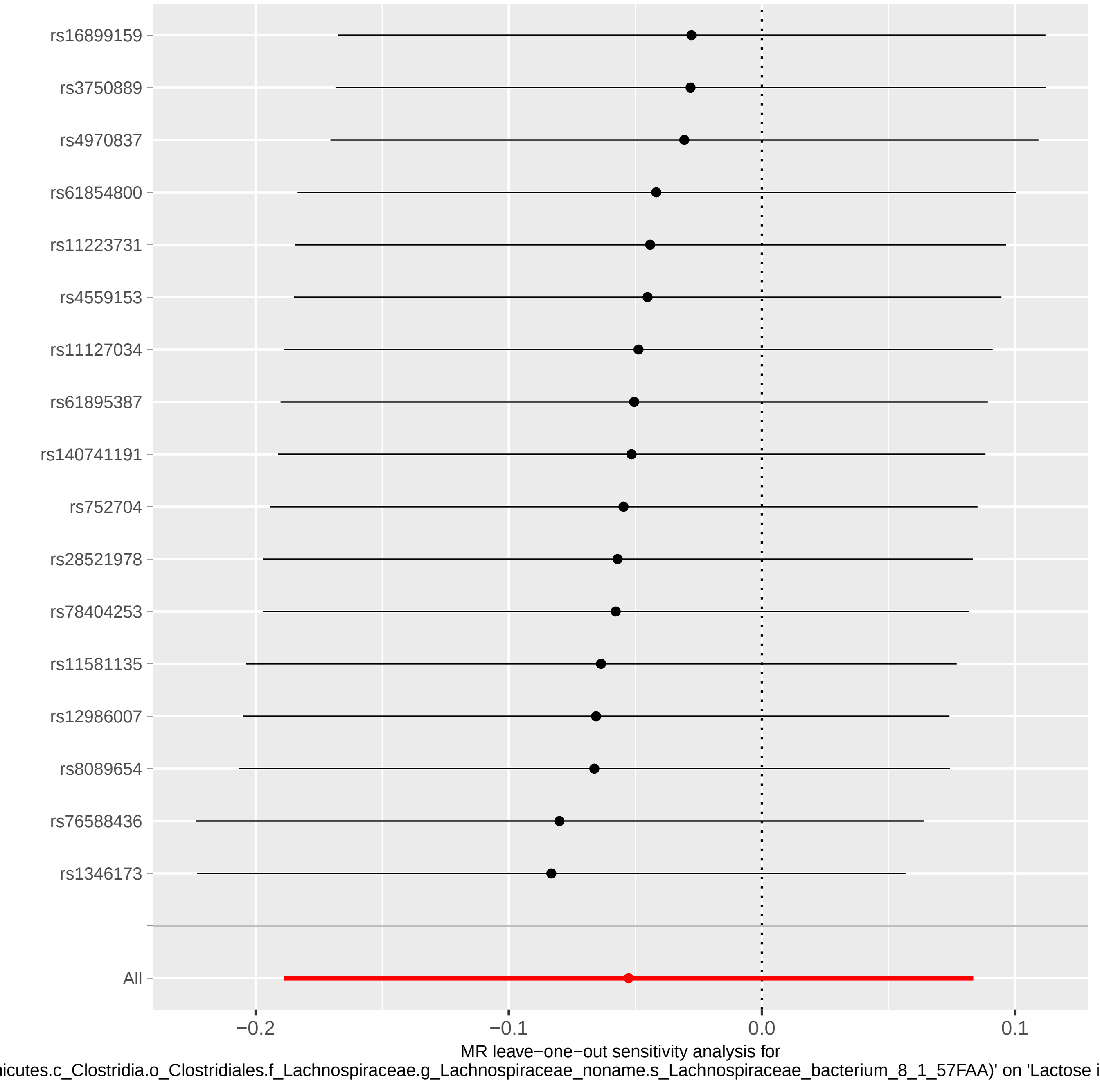
rs16899159
rs3750889
rs4970837
rs61854800
rs11223731
rs4559153
rs11127034
rs61895387
rs140741191
rs752704
rs28521978
rs78404253
rs11581135
rs12986007
rs8089654
rs76588436
rs1346173
All
−0.2
−0.1
0.0
0.1
MR leave−one−out sensitivity analysis for
nicutes.c_Clostridia.o_Clostridiales.f_Lachnospiraceae.g_Lachnospiraceae_noname.s_Lachnospiraceae_bacterium_8_1_57FAA)' on 'Lactose i
